# Albumin is ubiquitously expressed in the dolphin body and upregulated by an extracellular albumin shortage

**DOI:** 10.1242/jeb.249752

**Published:** 2025-05-22

**Authors:** Kaho Saito, Fumiki Hanafusa, Daiki Inamori, Takuya Itou, Miwa Suzuki

**Affiliations:** ^1^ Laboratory of Aquatic Animal Physiology, Applied Life Sciences, Graduate School of Bioresource Sciences, Nihon University, 1866 Kameino, Fujisawa, Kanagawa, 252-0880, Japan; ^2^ Taiji Whale Museum, 2934-2 Taiji, Taiji-cho, Higashimuro-gun, Wakayama, 649-5171, Japan; ^3^ Nihon University Veterinary Research Center, 1866 Kameino, Fujisawa, Kanagawa, 252-0880, Japan

**Keywords:** Cetacea, Aquatic adaptation, Ischemia, Osmoregulation, Antioxidants

## Abstract

This study demonstrates that hepatocyte-specific albumin is ubiquitously expressed and the protein is produced in both common bottlenose dolphins *Tursiops truncatus* and striped dolphins *Stenella coeruleoalba*. Quantitative PCR revealed that albumin precursor mRNA is expressed in all 12 tissues examined, with the highest levels observed in the liver. Next, TA cloning using skin and liver of the two species confirmed that the deduced albumin animo acid sequences in the tissues are 100% identical within each species and slightly different between the species or between tissues in common bottlenose dolphin and cultured cells derived from the same species. In addition, western blot analysis showed positive bands of approximately 66 kDa in all tissues examined, as seen with serum albumin. Immunohistochemistry assays also revealed that albumin protein was distributed in most cells in each tissue. These results suggest that albumin protein, with the same sequence as the serum albumin synthesized in liver, may be ubiquitously expressed in the dolphin body. Furthermore, to clarify the function of albumin, we removed bovine serum albumin from culture medium. This enhanced albumin mRNA expression in the cells. Albumin protein with the specific dolphin sequence then began to be detected in the medium. These data strongly indicate that albumin may be synthesized in many tissue types and extracellularly secreted in the same manner as in the liver. Additionally, albumin expression is likely to be upregulated in the absence of albumin around the cells. Ubiquitous albumin expression may be essential for dolphin peripheral cells, possibly to supply albumin during dive-induced ischemia.

## Teaser

**Summary:** Albumin is ubiquitously expressed in dolphins. In cultured renal cells, expression is enhanced by albumin depletion from the medium, suggesting that albumin is critical for dolphin cells.

## INTRODUCTION

Albumin is the most abundant circulating protein, with a plasma concentration of 35–50 mg ml^−1^ in mammals, including aquatic mammals (Dierauf and Gulland, 2001; Peters, 1995). It exerts a wide variety of functions, such as body water retention, by maintaining plasma oncotic pressure, antioxidant effects and other factors (Evans, 2002). Serum albumin (SA) is generally synthesized in the hepatocytes and secreted into the blood (Evans, 2002). In the liver, it is expressed as preproalbumin, which contains a 24 amino acid-long signal peptide. The first 18 amino acids and the additional six amino acids of the signal peptide are removed in the rough endoplasmic reticulum and Golgi body, respectively, resulting in mature albumin (SA) that is secreted into circulation (Peters, 1995; Strauss et al., 1977). SA released from hepatocytes enters blood vessels through the sinusoid intracellular space, then can migrate to the extravascular compartment through several mechanisms. For example, SA can move through the sinusoidal or fenestrated capillary pores in the liver, pancreas, bone marrow, small intestine and adrenal gland (Nicholson et al., 2000; Peters, 1995). In addition, the receptor albondin distributed in the plasma membrane probably transcytoses SA to support its passage through vascular endothelial cells (Milici et al., 1987; Tiruppathi et al., 1997). Through these routes, 60% of SA is distributed in extravascular spaces, while the rest remains in the blood vessels (Peters, 1995).

Cetaceans (whales and dolphins) are adapted to the marine environment, but must cope with various physiological stressors. One regulatory mechanism that carries a particularly heavy burden is osmotic regulation. Surrounded by seawater, cetaceans cannot access freshwater and are prone to losing water from their bodies because of the osmotic pressure differences. However, these mammals prevent water loss in a variety of ways, including by reducing water evaporation from their skin, as they have no sweat glands (Lester-Jones and Costa, 2006). Additionally, their urine is more concentrated than that of terrestrial mammals (Birukawa et al., 2005; Suzuki and Ortiz, 2015). Another physiological challenge of aquatic life is handling oxidative stress. Marine mammals, including cetaceans, routinely dive to feed, rest and escape from predators (Zenteno-Savin et al., 2012). Breath holding during diving and the subsequent respiration cause ischemia and reperfusion in tissues, inducing reactive oxygen species (ROS) production and oxidative stress (Wu et al., 2018; Zenteno-Savin et al., 2012). To counter this, these animals have enhanced tissue antioxidant activity, resulting in no apparent negative impacts of the dive-induced ROS generation on their bodies (Elsner et al., 1998; Vázquez-Medina et al., 2006, 2007; Wilhelm Filho et al., 2002). In these situations, improving osmotic regulatory function and antioxidant capacity is essential. Albumin is a protein that can assist with such situations by maintaining colloid osmotic pressure and reducing ROS levels, by scavenging ROS at its free cysteine residue at position 34 (Anraku et al., 2013; Evans, 2002). Thus, albumin may support cetacean aquatic adaptation, in addition to the previously known physiological response mechanisms for preventing water loss and alleviating oxidative stress, as described above.

In dolphins, the water retention and antioxidant capacities of albumin may play roles not only in blood vessels but also in extravascular spaces. In addition to being surrounded by hyperosmotic liquid, dolphins have a plasma osmotic pressure that is up to 75 mOsm kg^−1^ higher than that of humans and can fluctuate widely (Ortiz, 2001). Albumin in and/or around the cells can potentially help prevent water loss in cells surrounded by such hypertonic fluid. Moreover, dolphin SA has a higher antioxidative capacity than human SA, by obtaining free thiol groups from two easily dissociable disulfide bonds, and is an effective antioxidant in the blood (Suzuki et al., 2020). Because ROS production due to ischemia and reperfusion is mainly caused by mitochondria, xanthine oxidase in cytosol and NADPH oxidase in endothelial cells (Chouchani et al., 2016; Harrison, 2002; Kleikers et al., 2012), albumin can possibly help scavenge ROS and avoid damaging biomaterials in and/or around cells.

Albumin is normally synthesized specifically in the liver, as described above, but some reports have shown that it is expressed in extrahepatic organs. For example, albumin expressed in bovine tracheal gland serous cells probably contributes to the innate immune system (Shamay et al., 2005), while albumin synthesized in mouse retinas may transport retinoids in the visual cycle during early postnatal life (Dodson et al., 2001). In contrast, no previous studies have investigated albumin expression patterns in marine mammals, in which this protein may exert beneficial effects. In this study, as a first step towards re-evaluating the physiological roles of albumin in marine mammals, we investigated the expression and distribution of albumin in common bottlenose dolphins, *Tursiops truncatus* and striped dolphins, *Stenella coeruleoalba*. In addition, to explore its function in peripheral cells, we examined changes in albumin expression levels in dolphin cells cultured in an albumin-depleted medium.

## MATERIALS AND METHODS

### Ethics

The tissue samples used in this study were collected from striped dolphins, *Stenella coeruleoalba* (Meyen 1833), and a common bottlenose dolphin, *Tursiops truncatus* (Montagu 1821) caught by the regional fishery in Taiji (Wakayama, Japan). Dolphin fisheries were licensed by Wakayama prefecture government, and annual quotas were allocated by the government of Japan based on the stock assessment by Japan Fisheries Research and Education Agency (Kanaji et al., 2021, 2023; Kanaji and Sasaki, 2024; Minamikawa, 2024). Biological samplings were conducted under cooperation with Japan Fisheries Research and Education Agency (Minamikawa, 2024). Blood samples were collected from common bottlenose dolphins in the Taiji Whale Museum. The protocols for blood sampling (approval number: AP19BRS094-2) and genetic experiments (approval number: 2018-BS-041-01) were approved by Nihon University.

### Samples

For expression analysis, 12 organs – the cerebrum, pituitary gland, heart, lung, liver, stomach, pancreas, spleen, kidney, adrenal gland, skin (epidermis, dermis and subcutaneous fat) and skeletal muscle – were obtained from a female common bottlenose dolphin (specimen ID: 17TI123; body length BL 229 cm) in January 2017. Twelve similar organs were sampled from three striped dolphins (specimen IDs: 23SK027 male, BL 169 cm; 23SK041 male, BL 214 cm; and 23SK040 female, BL 190 cm) in January 2023. Each tissue was sub-sampled and preserved in RNAlater^®^ (Thermo Fisher Scientific, Waltham, MA, USA) at −20°C. For striped dolphin samples, sub-samples were also frozen at −80°C, except for the pituitary gland, as well as fixed in 4% paraformaldehyde (Wako, Osaka, Japan). To test the albumin synthesis capacity in cells other than hepatocytes, TK-ST cells, a cell line derived from a female common bottlenose dolphin kidney (Tashiro et al., 2023), were used. The cells were maintained in DMEM (Wako) supplemented with 10% fetal bovine serum (FBS; Nichirei, Tokyo, Japan) and 100 units ml^−1^ penicillin, 100 µg ml^−1^ streptomycin and 0.25 µg ml^−1^ amphotericin B at 37°C and 5% CO_2_. In addition, to obtain SA to use as a positive control for mature albumin, blood samples were collected from a captive female common bottlenose dolphin in the Taiji Whale Museum (Wakayama, Japan) in January 2020. The obtained whole-blood sample was centrifuged to retrieve the sera, then serum samples were frozen at −80°C until use.

### Preproalbumin (albumin precursor) mRNA expression analysis

#### Quantitative gene expression analysis in organs

To examine the albumin expression patterns in tissues of common bottlenose dolphin and striped dolphin and cultured cells derived from a common bottlenose dolphin, reverse transcription-quantitative PCR (RT-qPCR) was performed using the organ sub-samples preserved in RNAlater^®^ with the QuantiTect SYBR^®^ Green PCR Kit (Qiagen, Hilden, Germany) or KAPA SYBR Fast qPCR Kit (Nippon Genetics, Tokyo, Japan). The organ sub-samples and TK-ST cells were lysed, then total RNA was extracted using Isogen (Nippon Gene, Tokyo, Japan) following the manufacturer's suggested procedures. Next, cDNA was synthesized by reverse transcription of the total RNA extracted from each organ or TK-ST cells using the High-capacity RNA-to-cDNA kit (Thermo Fisher Scientific). PCR was performed with primer sets specific for the common bottlenose dolphin albumin and TATA-box binding protein (TBP) genes (Albumin-FW1 and Albumin-RV1, TBP-FW and TBP-RV; Table 1) using Roter-Gene Q (Qiagen) and the following conditions: initial denaturation at 95°C for 15 min and 30 cycles of denaturation at 95°C for 30 s, annealing at 60°C for 20 s, and extension at 72°C for 20 s for preproalbumin; initial denaturation at 95°C for 3 s and 40 cycles of denaturation at 95°C for 3 s, annealing at 53.8°C for 20 s, and extension at 72°C for 20 s for TBP. The preproalbumin mRNA levels were normalized to those of TBP, then the relative expression levels were compared among tissues. The PCR products were verified using 1% agarose gel electrophoresis with SYBR™ Safe DNA Gel Stain (Thermo Fisher Scientific) and observed on a Gel Doc™ EZ Imager (Bio-Rad, Hercules, CA, USA).

**Table 1.** Primer sequences

Primer	Sequence (5′–3′)
Albumin-FW1	TCCTGTGCTGAAGACTATCTGTCCTTG
Albumin-RV1	GATTTGTTTCTCATTCTCGGGAAGTGTGC
TBP-FW	AATCCCAAGCGGTTTGCTGC
TBP-RV	GGAACTTCACATCGCAGCTC
Albumin-FW2	GATTCTATTCATCCTACCTTTTCTCTTC
Albumin-RV2	ACAGATGAATAAGCTCTGAGTTTTC

#### Determination of full-length coding region expression

Albumin precursor expression was confirmed by RT-PCR analysis of liver and skin tissues and TK-ST cells with a specific primer set designed to amplify the full-length albumin precursor coding region (Albumin-FW2 and Albumin-RV2; Table 1). The cDNA samples described above were used. The PCR conditions were as follows: initial denaturation of 2 min at 94°C followed by 40 cycles of denaturation at 94°C for 30 s, annealing at 53°C for 30 s, and extension at 68°C for 1 min and 45 s. The PCR products were sequenced via TA cloning according to the method of Suzuki et al. (2016). The deduced albumin amino acid sequences were obtained by using the All-in-One Seq Analyzer (version 1.37, https://websites.umich.edu/~ino/blast.html) and aligned among samples.

### Protein assays

#### Western blot analysis

Albumin protein expression levels were examined by western blot analysis in TK-ST cells (derived from a common bottlenose dolphin) and the 11 striped dolphin organ tissue types stored at −80°C. TK-ST cells were cultured in a 75 cm^2^ flask and rinsed three times with ice-cold PBS to remove medium-derived SA. The cells were lysed by incubation with 2 ml of RIPA buffer (Thermo Fisher Scientific) containing Protease Inhibitor Cocktail Set III DMSO Solution (Wako) for 5 min on ice. The lysates were transferred to microtubes and centrifuged at 14,000 ***g*** and 4°C for 15 min, then the supernatants were collected as protein solutions. The protein concentration of each solution was measured using the Pierce™ Dilution-Free™ Rapid Gold BCA Protein Assay (Thermo Fisher Scientific). For the organ samples, total protein was extracted from 100 mg of each frozen tissue. Each tissue was cut into small pieces and rinsed five times with ice-cold PBS to remove as much blood-derived SA as possible, followed by homogenization with 1 ml of RIPA buffer containing the protease inhibitor cocktail. Following incubation for 30 min on ice, the homogenate was centrifuged at 14,000 ***g*** and 4°C for 20 min. The supernatant was retrieved, then the protein concentration was measured using a NanoDrop instrument (Thermo Fisher Scientific). As a positive control, SA was extracted from common bottlenose dolphin sera using the Pierce™ Albumin Depletion Kit (Thermo Fisher Scientific) according to Suzuki et al. (2020). The protein concentration of the extract was measured using the BCA Protein Assay kit.

Laemmli sample buffer (4× Sample buffer, Bio-Rad) containing 8% dithiothreitol (DTT) was added to the cell lysates, tissue protein extracts and extracted SA solution, then each mixture was heated at 75°C for 15 min for linearization and SDS binding. Each sample was separated using sodium dodecyl sulfate-polyacrylamide gel electrophoresis (SDS-PAGE) with a 5–20% gel (SuperSep™ Ace, Wako), then transferred to a polyvinylidene difluoride membrane (Bio-Rad). The membrane was washed with PBST and incubated in StartingBlock™ Blocking Buffer (Thermo Fisher Scientific) at room temperature for 30 min. Then, the membrane was incubated with 1:1000 dilution of a rabbit anti-bovine albumin antibody (A10-127A, Bethyl Laboratories, Montgomery, TX, USA) against whole bovine albumin overnight at 4°C. After washing with PBST, the membrane was incubated with undiluted EnVision+ Dual link System-HRP (K4063, Dako, Carpinteria, CA, USA) or 1:5 dilution of goat anti-rabbit IgG H&L (HRP polymer) (Abcam, Cambridge, UK) at room temperature for 30 min. After washing with PBST, positive reactions were visualized using 0.2 mg ml^−1^ 3,3′-diaminobenzidine (DAB) with 0.03% hydrogen peroxide (H_2_O_2_).

#### Immunohistochemistry

Albumin protein localization in tissues was determined by immunohistochemistry (IHC) assays using a modification of the method of Suzuki et al. (2008). Briefly, fixed tissues were embedded in paraffin, sectioned at 5 µm thickness and affixed on a glass slide. After being deparaffinized and washed, each section was immersed in 0.6% H_2_O_2_ in methanol for 30 min to inactivate endogenous peroxidases and then washed with water. After blocking with StartingBlock™ Blocking Buffer (Thermo Fisher Scientific) for 1 h, each section was incubated overnight at 4°C with a 1:250 dilution of the anti-albumin primary antibody mentioned above or a normal rabbit IgG (148-09551, Wako) as a negative control. After washing, undiluted goat anti-rabbit IgG H & L (HRP polymer) (ab214880, Abcam) was added to the section and incubated at room temperature for 30 min. After washing, positive reactions were visualized by immersing the section in 0.02% DAB with 0.03% H_2_O_2_.

### Albumin secretion assay

#### Detection of albumin in TK-ST cell culture medium

To confirm whether albumin synthesized in extrahepatic tissues is extracellularly secreted, albumin mRNA and protein expression, and albumin in medium were detected using TK-ST cells (derived from a common bottlenose dolphin) cultured in albumin-free medium. The experimental design was as follows: time points were set at 0 h to obtain the initial values before complete removal of albumin from the medium, and at 12, 24 and 48 h after removal to observe the time-course changes. The sample size was *n*=5 at each point. Experiments were conducted separately for the analysis of mRNA expression and the detection of intracellular and extracellular protein.

TK-ST cells were cultured in low-albumin medium [1:1 DMEM:HAM's F-12 (both from Wako) supplemented with 1% FBS (Nichirei), 100 units ml^−1^ penicillin, 100 µg ml^−1^ streptomycin and 0.25 µg ml^−1^ amphotericin B]. The cells were seeded into 25 cm^2^ flasks at a density of 5.0×10^5^ cells per flask in the low-albumin medium. After 36 h, the cells for the 0 h time point were collected. The cells in the remaining flasks were washed three times with PBS, then albumin-free medium [1:1 DMEM:HAM's F-12 containing 1 µg ml^−1^ human recombinant insulin (Wako), 0.5 mg ml^−1^ l(+)-glutamine (Wako), 100 units ml^−1^ penicillin, 100 µg ml^−1^ streptomycin and 0.25 µg ml^−1^ amphotericin B; Shamay et al., 2005] was added to the flasks. For mRNA expression analysis, the cells were lysed using Isogen. Total RNA was extracted from the cell lysate, then RT-qPCR analysis was performed for preproalbumin and TBP, as described previously. For intracellular and extracellular albumin protein detection, the medium was collected first and then the cells were lysed using RIPA buffer containing the protease inhibitor cocktail. The collected medium was centrifuged at 3000 ***g*** and room temperature for 5 min to precipitate dead cells. The supernatant and 5 ml of preculture medium were filter concentrated using a 30 kDa filter (Merk, Darmstadt, Germany). The protein concentration of the concentrated media and protein extracts from the cells were determined and the samples were subjected to western blot analysis for albumin protein levels using the methods described above. For the detection of an internal standard for cell lysates, 1:1000 dilution of anti-β-actin mAb (M177-3, Medical & Biological Laboratories, Tokyo, Japan) and 1:5 dilution of goat anti-mouse IgG H&L (HRP polymer) (ab214879, Abcam) were used. Positive reactions were visualized by a chemiluminescence technique using ImmunoStar^®^ Zeta (Wako) and the band intensities were analyzed using Image Lab™ software v.6.0 (BioRad) and shown as relative band intensities to the far-left band in each membrane. We conducted western blotting separately for the samples before and after the removal of albumin, because the protein abundance of the cell lysate after removal was too faint to detect under the same amount of applied protein and conditions as the samples before removal. Band intensities were compared among the samples only after albumin removal.

#### Detection of dolphin-derived albumin using liquid chromatography-tandem mass spectrometry (LC-MS/MS)

To demonstrate that TK-ST cells synthesize albumin that is distinguishable from FBS in the culture medium, LC-MS/MS analysis was applied. TK-ST cells cultured in the low-albumin medium mentioned above were seeded into two 75 cm^2^ flasks and cultured in this medium until they were 80% confluent. After washing the cells with PBS, 10 ml of albumin-free medium was added to each flask. After a 24 h incubation, the medium was collected, ultrafiltered using a 30 kDa filter (Merk), and replaced with the binding/wash buffer included in the Pierce™ Albumin Depletion Kit (Thermo Fisher Scientific). Then, albumin was extracted from the solution using this kit according to the manufacturer's suggested protocol. The eluted albumin-containing fraction was concentrated with a 3 kDa filter (Merk), then the protein concentration of the resulting solution was measured using the general Bradford protein assay method with Bio-Rad protein assay dye reagent (Bio-Rad). The albumin solution was mixed with Laemmli sample buffer (Bio-Rad) containing 8% DTT and heated at 75°C for 15 min. Then, 5 μg of each protein sample was separated by SDS-PAGE, as described above, and the gel was stained using the Silver Stain MS Kit (Wako) following the manufacturer's manual. The band at the expected molecular weight of dolphin SA (66 kDa; Fig. S1) was cut out from the gel and minced.

LC-MS/MS was conducted according to the method of Kina et al. (2021). Briefly, the minced gel containing the albumin fraction was destained and disulfide bonds were dissociated with DTT, followed by alkylation with iodoacetamide. After washing, the gel was digested with trypsin. Peptides were extracted with extraction solution (50% acetonitrile, 5% trifluoroacetic acid), dried, and redissolved with 0.1% formic acid. The peptide solution was used for LC-MS/MS analysis using a Q Exactive™ mass spectrometer (Thermo Fisher Scientific) equipped with a captive spray ion source and interfaced online with an Avance UHPLC system (Michrom Bioresources, Auburn, CA, USA). The obtained spectrum data were analyzed using SequestHT and Percolator nodes in Proteome Discover 2.5 (Thermo Fisher Scientific). The deduced amino acid sequence of the mature albumin protein expressed in TK-ST cells was used as a reference to detect dolphin albumin. The obtained sequence was verified to determine whether it was specific to dolphin albumin rather than bovine (*Bos taurus*) albumin (NP_851335.1).

### Statistical analyses

Differences in the relative preproalbumin mRNA expression levels among tissue samples were tested by linear mixed-effects models with animal ID as a random effect using the lme4 package, followed by a multiple comparison test among tissues using the emmeans package in R. Changes in preproalbumin mRNA expression levels and albumin protein band intensities in the intracellular and extracellular fractions over time after albumin removal were examined by Steel–Dwass multiple comparison test. The significance was set at 0.05 in all statistical analyses.

## RESULTS

### Albumin is expressed ubiquitously in the dolphin body and cultured cells

**Fig. 1.**
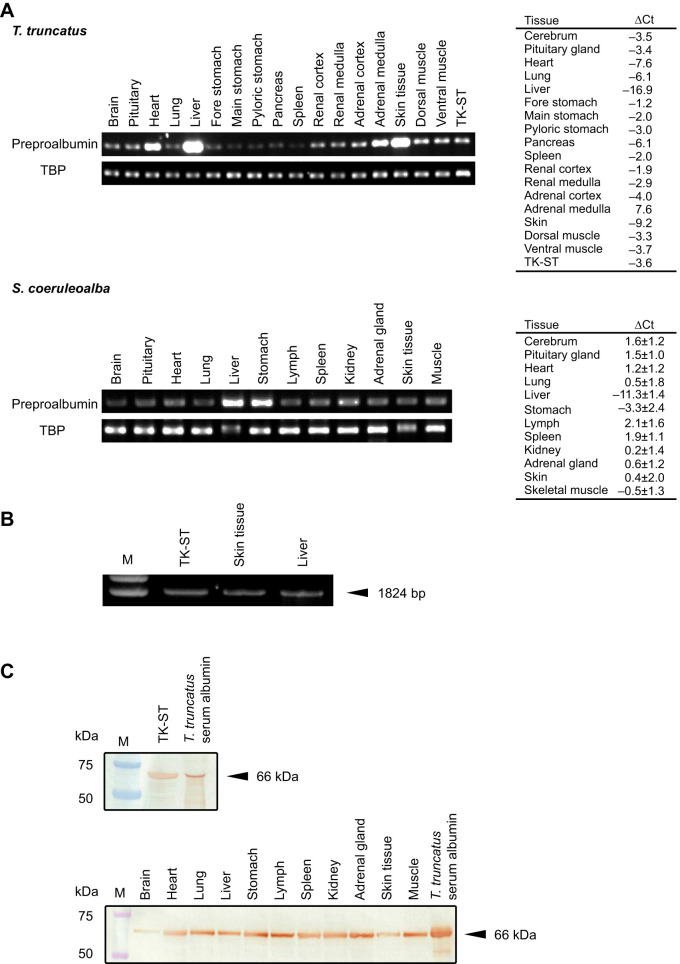
**Albumin expression patterns in common bottlenose dolphin and striped dolphin.** (A) Preproalbumin (albumin precursor) mRNA expression patterns in 12 common bottlenose dolphin (*Tursiops truncatus*) organs, cultured common bottlenose dolphin renal cells (TK-ST) and 12 striped dolphin (*Stenella coeruleoalba*) organs. The upper and lower panels for each species show the qPCR products for preproalbumin (227 bp) and TATA-box binding protein (TBP; 223 bp), respectively. The tables show mean±s.e.m. ΔCt (TBP−preproalbumin). (B) Expression of the full-length coding sequence of preproalbumin in TK-ST cells and striped dolphin skin and liver tissues as obtained by RT-PCR. (C) Western blot images of albumin protein expression in TK-ST cells (top) and 11 striped dolphin organs (bottom). Serum albumin from a common bottlenose dolphin was used as a positive control. M, molecular marker.

To confirm the albumin expression patterns in the body of dolphins, RT-qPCR for preproalbumin was conducted for the 12 common bottlenose dolphin and striped dolphin organs and for TK-ST cells (derived from a common bottlenose dolphin). As shown in Fig. 1A, a PCR product was obtained in all organs of both species, with the highest abundance observed in the liver, as well as cultured cells. The relative preproalbumin mRNA expression level normalized to that of TPB in each organ and its percentage relative to that in the liver in common bottlenose and striped dolphins are shown in Tables S1 and S2, respectively. The relative expression level in the liver of striped dolphin was significantly higher than that in each of the other tissues tested. In common bottlenose dolphin, the skin showed the second highest level at 0.41% that of the liver (Table S1), and in striped dolphin the stomach was the second highest with 2.50±5.13% (Table S2).

As shown in Fig. 1B, the same amplification size of the full-length albumin precursor coding sequence was obtained in the liver and skin of striped dolphins and in TK-ST cells derived from common bottlenose dolphin. The size was also the same in the liver and skin of common bottlenose dolphin (data not shown). The sequences were identified as preproalbumin. The deduced amino acid sequences of the liver and skin were identical within each species, while the sequence was 98.4% identical between common bottlenose dolphin and striped dolphin and 99.7% identical between common bottlenose dolphin tissues and TK-ST cells (Fig. S2).

The albumin protein expression detected by western blot analysis in the TK-ST cells and in tissues from 11 organs of striped dolphin is shown in Fig. 1C. The same 66 kDa target band was detected in the cells and all tissues (Fig. 1C) as in the extracted SA from common bottlenose dolphin.

### Albumin protein is diversely distributed in peripheral tissues

**Fig. 2.**
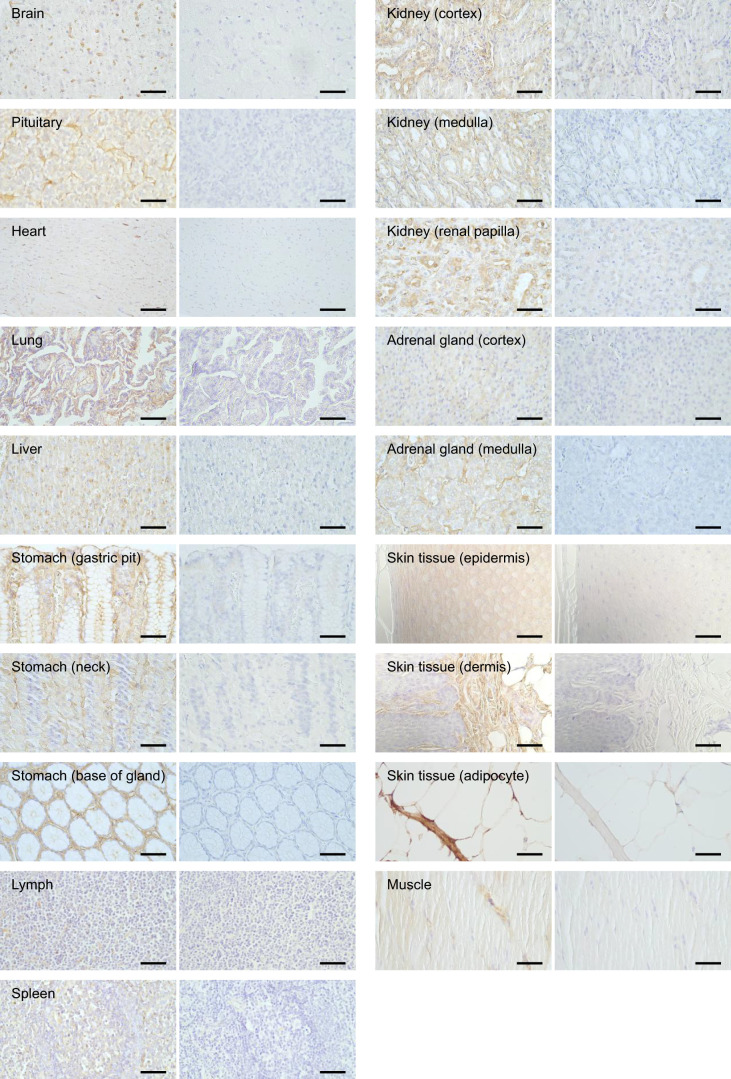
**Immunohistochemistry (IHC) assays for albumin protein in striped dolphin organ tissues.** IHC staining was conducted with an anti-bovine albumin primary antibody (left) or rabbit IgG negative control (right). Scale bars: 50 µm.

To determine albumin protein localization in tissues, IHC assays were performed using an anti-albumin antibody in 12 organ tissues of striped dolphins. In the liver tissue, which was used as a positive control, positive albumin staining was obtained in the hepatocytes (Fig. 2). Albumin protein was also detected in most cells in the other 11 organs, although the staining was faint in the brain and heart tissues (Fig. 2). Intensely positive albumin IHC staining was observed in the stomach, especially in the surface mucous cells and parietal cells, compared with that in other tissues (Fig. 2). In the kidney, positive albumin staining was obtained in the renal tubule, with stronger staining observed in the medulla collecting ducts (Fig. 2).

### Albumin produced in TK-ST cells is extracellularly secreted and upregulated in response to a shortage of surrounding SA

**Fig. 3.**
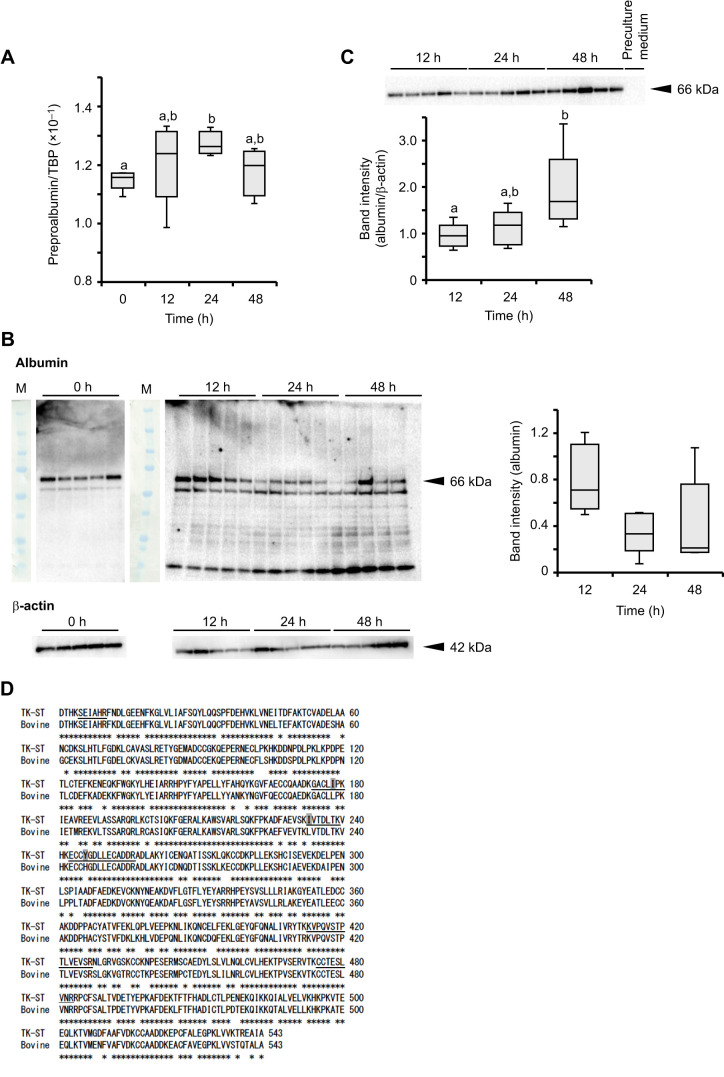
**Changes in intracellular and extracellular albumin expression levels in cultured common bottlenose dolphin renal cells (TK-ST) with depletion of pericellular albumin.** (A) Preproalbumin mRNA expression levels relative to those of TATA-box binding protein (TBP) in cells before (0 h) and 12, 24 and 48 h after albumin removal from the culture medium (*n*=5). (B) Western blot image of albumin and β-actin protein expression before (0 h) and 12, 24 and 48 h after albumin removal. M, molecular marker. The distribution of band intensities for albumin normalized to that of β-actin in the cells is shown as box plots (*n*=5). (C) Western blot image of albumin in the cell culture medium 12, 24 and 48 h after albumin removal and in the preculture medium. Distributions of band intensities for albumin are shown as box plots (*n*=5). (D) Amino acid sequence alignment of the deduced mature albumin secreted from TK-ST cells and bovine serum albumin (NP_851335.1). Asterisks indicate conservation of sequence between species. Underlines indicate the obtained sequence extracted from the TK-ST cell culture medium by LC-MS/MS. The amino acids shaded in gray are the residues in the detected sequences that differ between common bottlenose dolphin and bovine serum albumin. Box plots show median, first and third quartiles, minimum and maximum. Different letters indicate a significant difference in the means (*P*<0.05).

To examine whether albumin synthesized in extrahepatic tissues is extracellularly secreted, changes in the albumin content of the intracellular and extracellular fractions, as well as the mRNA expression levels, were analyzed using TK-ST cells (derived from a common bottlenose dolphin). The relative expression levels (preproalbumin/TBP) were significantly increased 24 h after albumin removal (0.13±0.063, mean±s.e.m.) from the medium compared with that before the removal (0.11±0.058) (*P*=0.04) (Fig. 3A). There were no significant differences over time for intracellular albumin protein, and the intensities of small molecule bands increased 48 h after albumin removal (Fig. 3B). In the medium, albumin was not detected before cell culture, but was observed at 12, 24 and 48 h (Fig. 3C). The extracellular albumin was significantly increased at 48 h compared with that at 12 h (Fig. 3C; *P*=0.04). The protein sequence of the albumin secreted from the TK-ST cells was analyzed by LC-MS/MS. As shown in Fig. 3D, the peptide fragments obtained were identified as mature dolphin albumin, which included the characteristic sequences only seen in cetaceans.

## DISCUSSION

This study revealed that albumin is widely expressed in the bodies of the common bottlenose dolphin and striped dolphin. We also showed that albumin expression and secretion were enhanced when dolphin cells (derived from a common bottlenose dolphin) were cultured in the absence of extracellular albumin. These data suggest that albumin may be essential for the peripheral cells in dolphins.

According to our RT-qPCR data of a common bottlenose dolphin and striped dolphins, western blots and IHC data of striped dolphins, albumin is ubiquitously expressed in the dolphin body, although it is primarily produced in the liver, as in other mammals. The deduced preproalbumin primary protein sequence was identical between tissues within each species. The 1.6% sequence difference among dolphin species is probably due to species differences and the 0.3% difference between common bottlenose dolphin and cultured cells originating from the same species may be caused by the repeated passage of TK-ST cells. In addition, when TK-ST cells were cultured in an albumin-free medium, albumin protein was detected in both the intracellular and extracellular fractions and it possessed the protein sequence unique to TK-ST cells (derived from a common bottlenose dolphin). These experiments suggest that all organs in the dolphin body express albumin and secrete it extracellularly, as occurs in the liver in common bottlenose dolphin and striped dolphin.

The RT-qPCR results of striped dolphins suggest that albumin synthesis may also be active in the stomach. However, no significant differences were detected between these organs, mainly because of the large deviation among individuals. Stress alters protein expression; it increases the levels of inflammatory markers such as interleukin-6 and C-reactive protein (Jain et al., 2011). Inflammation also elevates acute-phase proteins (APPs), including C-reactive protein, while reducing negative APPs such as albumin (Steptoe et al., 2007). The dolphins used in this study were derived from driven fisheries and may have experienced stress from chase and capture. Whether albumin synthesized in dolphin multiple organs is a negative APP is unknown, but the condition may explain individual differences in albumin expression. Moreover, the albumin IHC staining patterns in the surface mucous cells and parietal cells in the stomach were relatively strong. Dolphins may ingest 12–13 ml kg^−1^ body mass of seawater per day as they feed (Hui, 1981), causing their gastrointestinal tract to be exposed to liquid with a higher salt content than their body fluids. Albumin largely contributes to maintaining colloid osmotic pressure, mainly from this protein being an osmolyte, as well as by the Donnan effect, which attracts positively charged molecules and keeps them within the blood vessels to retain water (Nicholson et al., 2000). Albumin expressed in each tissue type may play such roles locally and support water retention in the peripheral tissues of dolphins. The high albumin expression levels observed in the stomach may prevent water leakage from the body when the tissue is exposed to high salinity. In the kidney, albumin was particularly localized around the collecting ducts in the renal medulla. The renal medulla is one of the organs exposed to hyperosmotic liquid to concentrate urine (Woo and Kwon, 2002). Urine osmolality in dolphins is relatively higher than that of terrestrial mammals of the same size (Ortiz, 2001; Suzuki and Ortiz, 2015). Albumin within the cells of the collecting ducts may increase osmolarity to help prevent them from shrinking and reinforce water reabsorption from urine. In addition, albumin expression was higher in the skin, which is constantly exposed to seawater, and this may also contribute to water retention in cells.

Other organ tissues of striped dolphins, including the cerebrum, pituitary gland and heart, showed low albumin expression levels. This may be related to the antioxidant effect of albumin. When dolphins dive, blood flow is prioritized to the central nervous system and heart, which are sensitive to oxygen. Subsequently, blood flow to other peripheral tissues is dramatically reduced and can cause ischemia (Bevan and Butler, 1992; Kooyman and Ponganis, 1998). After surfacing, these ischemic tissues are re-oxidized and aerobic respiration restarts. This ischemia and reperfusion produce ROS, resulting in oxidative stress. In the mitochondrial system, the electron transport chain is disrupted during ischemia, leading to the accumulation of substrates that are oxidized all at once during reperfusion, resulting in ROS production (Chouchani et al., 2016). In the xanthine oxidoreductase system, xanthine dehydrogenase is converted to xanthine oxidase, which generates ROS during ischemia, and ROS production is further promoted by the re-entry of oxygen during reperfusion (Harrison, 2002). In the NADPH oxidase system, the expression of NADPH oxidase, which is enhanced during ischemia, leads to ROS production by oxidizing the oxygen that flows in during reperfusion (Kleikers et al., 2012). Marine mammals, including dolphins, have an improved antioxidant system in tissues to buffer this oxidative stress. SA has an antioxidant ability and is responsible for scavenging 70% of the ROS activity in blood (Bourdon and Blache, 2001). In human SA, free thiol at position 34 plays an important role by trapping ROS (Anraku et al., 2013, 2015). Despite the C34S substitution, which results in a 30% reduction of this ability in human SA (Anraku et al., 2015), dolphin SA has a higher antioxidant activity than human SA because of the extra free thiol residues obtained by dissociation of disulfide bonds (Suzuki et al., 2020). Albumin synthesized in peripheral tissues may contribute to scavenging in cells with high ROS production. This can explain the low albumin expression levels observed in the brain and heart tissues, as these organs do not experience ischemia and reperfusion and therefore do not strongly rely on the antioxidant ability of albumin.

In the albumin-free medium, cellular albumin expression levels increased 24 h after albumin removal. The extracellular albumin protein content in the medium did not change between 12 and 24 h and increased at 48 h. In contrast, intracellular albumin content did not change over time. As albumin is immediately secreted extracellularly upon synthesis (Rothschild et al., 1988), the active extracellular secretion may lead to this result. These data indicate that the albumin expression and its secretion may be enhanced when albumin is depleted in the pericellular space. Additionally, Fig. 3A shows an increase in expression from 12 h to 24 h after the removal, followed by a decrease at 48 h although no statistical differences were observed. The highest albumin protein level in the medium at 48 h is probably due to the low but present mRNA expression at that time. The removal of albumin from the culture medium markedly reduced the intracellular albumin concentrations, and as a result, albumin protein could not be detected under the same western blotting conditions as before the removal. In this regard, it should be noted that the cells may ingest albumin when FBS is included in the culture medium. In the human renal proximal tubules, albumin filtered through the glomeruli is reabsorbed by the megalin and cubilin receptors via endocytosis (Bern et al., 2015). Tumor cells also take in albumin as a nutrient through micropinocytosis (Kamphorst et al., 2015). Because the TK-ST cells were derived from epithelial cells (Tashiro et al., 2023), these cells may also take in albumin as a nutrient. However, the increase or decrease in intracellular albumin levels after the culture medium was replaced with albumin-free medium requires further investigation.

Multiple bands were enhanced in cell lysates after albumin removal. Suzuki et al. (2020) suggested that dolphin albumin has easily dissociable disulfide bonds and may be degraded by oxidative stress or other factors. Considering that the multiple bands were located under 66 kDa, these bands are probable albumin degradation products. In cetaceans, the respiratory quotient is around 0.71–0.75, indicating they use fat and protein as energy substrates (Suzuki and Ortiz, 2024). Albumin-free medium does not contain FBS and fat; thus, the cells should have used glucose and glutamine, depleting them by the end of the assay. The intense bands at around 10 kDa observed at 48 h might be due to nutrient depletion in the culture medium, leading cells to enhance albumin degradation through autophagy.

Taken together, we showed ubiquitous expression of albumin in dolphin body and enhanced expression and secretion levels when albumin was depleted in the pericellular space. These findings indicate that albumin is essential for cell survival in dolphins. In terrestrial mammals, 60% of SA migrates from the blood into the interstitium (Peters, 1995). However, when dolphins dive, ischemia prevents albumin from being supplied to peripheral tissues; thus, these tissues must produce albumin locally to regulate osmolarity and redox homeostasis. Considering such physiological and ecological features, we postulate that the ubiquitous and flexible expression of albumin can support dolphin adaptation to aquatic life.

### Limitations

This is the first report to investigate albumin expression patterns in multiple marine mammal organs. However, the small sample size (*N*=3 dolphins) is one limitation of this study. Dolphin health and/or stress condition may affect albumin expression. By increasing the number of samples analyzed, the large expression level deviations observed between individuals can be addressed and more universal results can be obtained. In addition, albumin was detected in various tissues using western blot and IHC. However, it is uncertain whether this albumin was synthesized in each tissue or not, as it is impossible to completely remove blood from the tissues. To more accurately assess albumin synthesis in cells, synthetic quantity evaluation using labeled amino acids is needed in the future.

### Conclusions

This study revealed that albumin is ubiquitously expressed in the body of dolphins. The expression and secretion levels increased in cultured renal cells when albumin was eliminated from the medium. These results suggest that albumin may be essential for dolphin cells. Its osmoregulatory action and antioxidant ability may play critical roles in dolphins, which live in hyperosmotic environments and experience dive-induced oxidative stress. Further studies on albumin functions in peripheral tissues and its expression regulation mechanisms are needed to elucidate how albumin is involved in dolphin adaptation to aquatic environments.

## Supplementary Material

10.1242/jexbio.249752_sup1 Supplementary information
