# Supplementary material for: Albumin is ubiquitously expressed in the dolphin body and upregulated by an extracellular albumin shortage
Source: J Exp Biol. 2025 May 22;228(10):jeb249752. doi: 10.1242/jeb.249752 (PMC12148036; doi:10.1242/jeb.249752)
Supplement: Supplementary information [file jexbio-228-249752-s1.pdf]

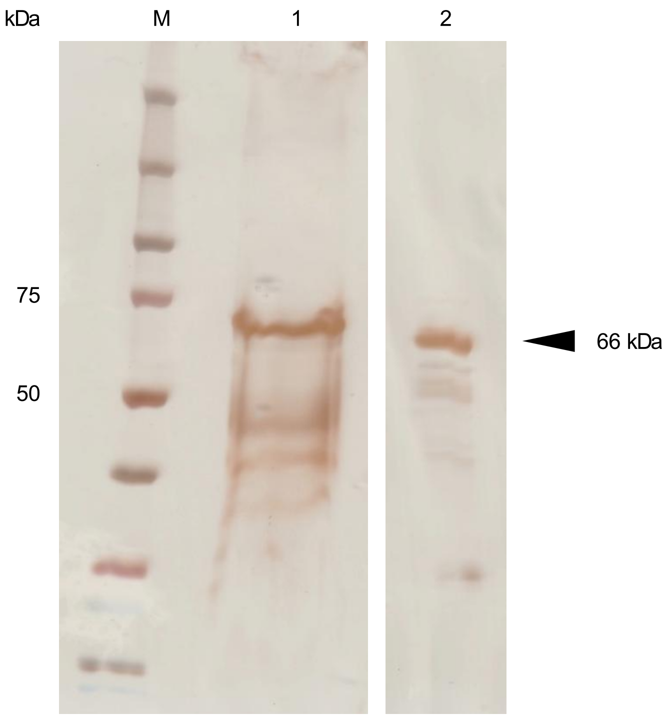

Fig. S1. Images of SDS-PAGE gel after silver staining for albumin extracted from the cultured medium with common bottlenose dolphin renal cell (TK-ST) (lane no. 1) and serum albumin of common bottlenose dolphin (lane no. 2). The samples were electrophoresed on the same gel. M: marker.

```
T. truncatus    MKWVTFISLIFLFSSAYSRGVFRRDTHKSEIAHRFNDLGEENFKGLVLIAFSQYLQQSPF 60
TK-ST cell      MKWVTFISLIFLFSSAYSRGVFRRDTHKSEIAHRFNDLGEENFKGLVLIAFSQYLQQSPF 60
S. coeruleoalba MKWVTFISLIFLFSSAYSRGVFRRDTHKSEIAHRFNDLGEENFKGLVLIAFSQYLQQSPF 60
                ************************************************************

T. truncatus    DEHVKLVNEITDFAKTCVADESAANCDKSLHTLFGDKLCAVASLRETYGEMADCCGKQEP 120
TK-ST           DEHVKLVNEITDFAKTCVADESAANCDKSLHTLFGDKLCAVASLRETYGEMVDCCGKQEP 120
S. coeruleoalba DEHVKLVNEITDFAKTCVADESAANCDKSLHTLFGDKLCAVASLRETYGEMADCCGKQDP 120
                *************************************************** ****** *

T. truncatus    ERNECLLKHKDDNPDLPKLKPDPETLCTEFKENEQKFWGKYLHEIARRHPYFYAPELLYF 180
TK-ST           ERNECLLKHKDDNPDLPKLKPDPETLCTEFKENEQKFWGKYLHEIARRHPYFYAPELLYF 180
S. coeruleoalba ERNECLLKHKDDNPDLPKLKPDPETFCTEFKENEQKFWGKYLYEIARRHPYFYAPELLYF 180
                ************************* **************** *****************

T. truncatus    AHQYKGVFAECCQAADKGACLIPKIEAVREEVLASSARQRLKCTSIQKFGERALKAWSVA 240
TK-ST           AHQYKGVFAECCQAADKGACLIPKIEAAREEVLASSARQRLKCTSIQKFGERALKAWSVA 240
S. coeruleoalba AHQYKGVFAECCQAADKGACLIPKIETVREEVLASSARQRLKCTSIQKFGERALKAWAVA 240
                **************************  ***************************** **

T. truncatus    RLSQKFPKADFAEVSKIVTDLTKVHKECCYGDLLECADDRADLAKYICENQATISSKLQK 300
TK-ST           RLSQKFPKADFAEVSKIVTDLTKVHKECCYGDLLECADDRADLAKYICENQATISSKLQK 300
S. coeruleoalba RLSQKFPKADFAEVSKIVTDLTKVHKECCHGDLLECADDRADLAKYICENQATISSKLQK 300
                **************************** *******************************

T. truncatus    CCDKPLLEKSHCISEVEKDELPENLSPIAADFAEDKEVCKSYNEAKDVFLGTFLYEYARR 360
TK-ST           CCDKPLLEKSHCISEVEKDELPENLSPIAADFAEDKEVCKSYNEAKDVFLGTFLYEYARR 360
S. coeruleoalba CCDKPLLEKSHCISEVEKDELPENLSPIAADFAEDKEVCKNYNEAKDVFLGTFLYEYARR 360
                **************************************** *******************

T. truncatus    HPEYSASLLLRIAKGYEATLEDCCAKDDPPACYAAVFEKLQPLVEEPKNLIKQNCELFEK 420
TK-ST           HPEYSASLLLRIAKGYEATLEDCCAKDDPPACYAAVFEKLQPLVEEPKNLIKQNCELFEK 420
S. coeruleoalba HPEYSVSLLLRIAKGYEATLEDCCAKDDPPACYATVFEKLQPLVEEPKNLIKQNCELFEK 420
                ***** *************************** **************************

T. truncatus    LGEYQFQNALIVRYTKKVPQVSTPTLVEVSRNLGRVGSKCCKNPESERMSCAEDYLSLVL 480
TK-ST           LGEYQFQNALIVRYTKKVPQVSTPTLVEVSRNLGRVGSKCCKNPESERMSCAEDYLSLVL 480
S. coeruleoalba LGEYQFQNALIVRYTKKVPQVSTPTLVEVSRNLGRVGSKCCKNPESERMSCAEDYLSLVL 480
                ************************************************************

T. truncatus    NQLCVLHEKTPVSERVTKCCTESLVNRRPCFSALTVDETYEPKAFDEKTFTFHADLCTLP 540
TK-ST           NQLCVLHEKTPVSERVTKCCTESLVNRRPCFSALTVDETYEPKAFDEKTFTFHADLCTLP 540
S. coeruleoalba NRLCVLHEKTPVSERVTKCCTESLVNRRPCFSALTVDETYEPKAFDEKTFTFHADLCTLP 540
                * **********************************************************

T. truncatus    ENEKQIKKQIALVELVKHKPKVTEEQLKTVMGDFAAFVDKCCAADDKEPCFALEGPKLVV 600
TK-ST           ENEKQIKKQIALVELVKHKPKVTEEQLKTVMGDFAAFVDKCCAADDKEPCFALEGPKLVV 600
S. coeruleoalba ENEKQIKKQIALVELVKHKPKVTEEQLKTVMGDFAAFVDKCCAADDKEPCFALEGPKLVV 600
                ************************************************************

T. truncatus    KTREAIA 607
TK-ST           KTREAIA 607
S. coeruleoalba KTREAIA 607
                *******
```

Fig. S2. An alignment of deduced amino acid sequencing of preproalbumin from the liver and skin of striped dolphin and cultured common bottlenose dolphin renal cell (TK-ST). Asterisk indicates same amino acid among samples.

Journal of Experimental Biology • Supplementary information

Table S1. Relative expression level of preproalbumin (albumin precursor) to TBP in 12 organs of common bottlenose dolphin (n = 1) and the percentage of expression in each organ to that in the liver.

organ	preproalbumin/TBP	percentage to that in liver (%)
liver	$1.17E^{+03}$	100.00
skin tissue	$4.78E^{+00}$	0.410
heart	$1.23E^{+00}$	0.106
pancreas	$5.75E^{-01}$	0.049
lung	$4.36E^{-01}$	0.037
TK-ST	$4.20E^{-01}$	0.036
adrenal medulla	$1.55E^{-01}$	0.013
adrenal cortex	$1.09E^{-01}$	0.009
brain	$7.84E^{-02}$	0.007
ventral muscle	$7.62E^{-02}$	0.007
pituitary	$6.18E^{-02}$	0.005
dorsal muscle	$5.61E^{-02}$	0.005
pyloric stomach	$5.52E^{-02}$	0.005
renal medulla	$4.32E^{-02}$	0.004
spleen	$2.48E^{-02}$	0.002
main stomach	$2.25E^{-02}$	0.002
renal cortex	$2.10E^{-02}$	0.002
fore stomach	$1.25E^{-02}$	0.001

Journal of Experimental Biology • Supplementary information

Table S2. Relative expression level of preproalbumin (albumin precursor) to TBP in 12 organs of striped dolphin (n = 3) and the percentage of expression in each organ to that in the liver. Data presented as mean ± s.e.m.

organ	preproalbumin/TBP	percentage to that in liver (%)
liver	$2.11E^{+03}$±$4.93E^{+01}$	100.00±8.28
stomach	$5.27E^{+01}$±$9.02E^{-01}$	2.50±5.13
skin tissue	$5.86E^{+00}$±$3.16E^{+00}$	0.28±0.44
lung	$1.67E^{+00}$±$1.65E^{+00}$	0.08±0.48
kidney	$8.94E^{-01}$±$9.94E^{-01}$	0.04±0.30
adrenal gland	$5.51E^{-01}$±$6.99E^{-01}$	0.03±0.21
muscle	$5.37E^{-01}$±$5.84E^{-01}$	0.03±0.34
heart	$2.91E^{-01}$±$4.99E^{-01}$	0.01±0.31
lymph	$2.65E^{-01}$±$5.58E^{-01}$	0.01±0.17
brain	$2.34E^{-01}$±$5.02E^{-01}$	0.01±0.15
pituitary	$2.19E^{-01}$±$3.69E^{-01}$	0.01±0.18
spleen	$1.66E^{-01}$±$3.24E^{-01}$	0.01±0.21

Journal of Experimental Biology • Supplementary information
